# Supplementary material for: Controllable assembly of sub-1 nm nanowires for the construction of aerogels
Source: Nat Commun. 2026 Mar 17;17:4053. doi: 10.1038/s41467-026-70713-8 (PMC13139467; doi:10.1038/s41467-026-70713-8)
Supplement: Supplementary file 1 — Supplementary Information [file 41467_2026_70713_MOESM1_ESM.pdf]

Supplementary Information

Controllable assembly of sub-1 nm nanowires for the construction of aerogel

Yuxiang Du[1,2], Yueyue Xiu[1,2], Xin Yang[1,2], Rui Fu[1,2], Dan Liu[1,2], Lipeng Liu[1,2], Huazheng Sai[1,2]*

[1]School of Chemistry and Chemical Engineering, Inner Mongolia University of Science & Technology, Baotou, 014010, China; [2]Aerogel Functional Nanomaterials Laboratory, Inner Mongolia University of Science & Technology, Baotou, 014010, China.

*Correspondence authors. E-mail: {HYPERLINK "mailto:shz15@tsinghua.org.cn"} (H.S.)

The file includes:

Supplementary Text

Supplementary Fig. S1-S17

References

Other Supplementary Materials for this manuscript include the following:

Supplementary Video S1-3 (.mp4)

Supplementary Text

Sample pretreatment before testing

For XPS analysis, ligand-exchanged SNWs initially dispersed in tert-butanol were purified through a series of precipitation-redispersion cycles. Briefly, acetonitrile was added to the dispersion as an antisolvent to induce the precipitation of SNWs, followed by centrifugation and removal of the supernatant. The obtained solids were then redispersed in tert-butanol and precipitated again with acetonitrile; this purification process was repeated three times to ensure the complete removal of residual ligands and impurities. The purified SNWs were subsequently dried in an electric thermostaticdrying oven at 90 °C for 6 h.

In a similar manner, pristine SNWs (without ligand exchange) originally dispersed in cyclohexane were prepared for testing by adding ethanol to trigger precipitation. The centrifugation and washing steps were performed three times to facilitate the thorough removal of cyclohexane, followed by drying at 90 °C for 6 h. The same pretreatment protocols were rigorously applied to all SNW samples prior to TGA and FTIR spectroscopy measurements to ensure consistency across characterizations.

Density calculation

The volume (v_c) of the SNWAs were measured by using a vernier caliper, and the mass (m_c) of the SNWAs were measured by using a measurement and analysis balance. The density of the SNWAs were calculated from the ratio of mass to volume (m_c/ v_c).

Molecular Dynamics (MD) simulations

Atomistic molecular dynamics simulations have been performed in the GROMACS (version 2020.6) simulation package, using the General Amber force field (GAFF2). The GdOOH nano-wire around 6 nm was built through replicating the crystal cell, and 21 15-HYDROXYPENTADECANOIC ACID were randomly placed around the nano-wire. The acid molecules adsorb onto the nanowire within 2 ns MD simulations. 4 such decorated nano-wire were then randomly placed in a cubic box of 10 nm, and 30 citric molecules and 56 H_3O^+ ions were randomly placed into the box. After thousands of steps of energy minimization, the system was solvated with 5676 TBA solvent molecules. Energy minimization was again performed for thousands of steps for the whole system and the solvent was equilibrated for 5 ns with restraints on the solute. Finally, the production run of 50 ns molecular dynamics simulation was performed for the decorated nano-wires to assemble with each other in the TBA solvent. The temperature was coupled to 298 K using the Nose-Hoover method and the pressure was coupled to 1 atm using the Parrinello-Rahman method. The cutoff scheme of 1.2 nm was implemented for the non-bonded interactions, and the Particle Mesh Ewald method with a fourierspacing of 0.1 nm was applied for the long range electrostatic interactions. All covalent bonds with hydrogen atoms were constraint using the LINCS algorithm[1-3].

Calculation of the Mass Proportion of Organic Compounds on SNWs

The thermal decomposition curves of OA and 15-HA have two organic thermal decomposition stages (Fig. S2). The first organic matter thermal decomposition stage exhibits rapid decomposition at 200-450 °C. The second organic matter thermal decomposition stage exhibits slow decomposition at 450-550 °C. As shown in the following equation, the mass of

organic matter in the two stages adds up to 100%.

$$m_A+m_B=100\% \qquad (S1)$$

Where m_A (85.41%) is the mass of rapidly thermally decomposing organic matter. m_B (14.59%) is the mass of slowly thermally decomposing organic matter.

From the thermal decomposition curves of SNWs, it can be seen that the rapid decomposition mass of organic matter (m_C) at 200-450 °C accounts for about 37.07%. At 450-550°C, there is still a small amount of slow thermal decomposition of organic matter (Fig. 2h). The proportion of small amount of organic matter (m_D) can be calculated according to the following equation:

$$\frac{m_A}{m_C}=\frac{m_B}{m_D} \qquad (S2)$$

$$m_D=\frac{m_C \times m_B}{m_A}=6.26\% \qquad (S3)$$

Where m_A equals 85.41%. m_B equals 14.59. m_C equals 37.07%.

The total mass m_E of organic matter on SNWs was obtained by adding ma and mb based on the following equation:

$$m_E=m_C+m_D=43.36\% \qquad (S4)$$

Where m_C equals 37.07%. m_D equals 6.26%

Calculation of the oleic acid content displaced by the ligand

Firstly, nine oleic acid standards with mass fraction of 0.1%, 0.2%, 0.3%, 0.35%, 0.4%, 0.45%, 0.5%, 0.6%, and 0.7% were prepared using gradient dilution method. Obtain the standard sample of oleic acid and the gas chromatogram of the sample through gas

chromatography-mass spectrometry. Then integrate the area of the oleic acid standard peak and perform linear fitting to obtain the following equation:

$$y=-2.31787E7+4.7639E8*x \quad \text{(S5)}$$

$$r^2=0.99108$$

Where y is the area of the oleic acid peak in the standard and x is the amount of oleic acid (%) in the standard.

Substitute the area of the oleic acid peak in the sample into the following formula to calculate the mass fraction of oleic acid in the sample:

$$x_1=\frac{y_1+2.31787E7}{4.7639E8}=0.56 \quad \text{(S6)}$$

Where y_1(2.44596E8) is the area of the oleic acid peak in the sample and x_1 is the amount of oleic acid (%) in the sample.

In an oleic acid solution, given the mass fraction of oleic acid and the mass of the solvent, the mass of oleic acid in the solution can be obtained according to the following formula:

$$\frac{m_1}{m_1+m_2}\times 100\%=x_1 \quad \text{(S7)}$$

$$m_1=0.032\ g$$

Where m_1 is the mass of oleic acid, the mass of solvent m_2 is obtained by weighing with an electronic precision balance. x_1 is the mass fraction of oleic acid.

The mass of oleic acid displaced by the ligand can be calculated using the equation above, based on the total mass of SNWs and ligands. Subsequently, the percentage of oleic acid undergoing ligand substitution relative to the total mass can be determined using the following

formula:

$$Wt_0\%=\frac{m_1}{m_0}\times100\%=42.7\% \qquad (S8)$$

Where m_1 (0.032 g) is the mass of oleic acid. m_0 is the mass of SNWs (0.075 g) obtained by weighing with an electronic precision balance. $Wt_0\%$ is the mass fraction of oleic acid on SNWs.

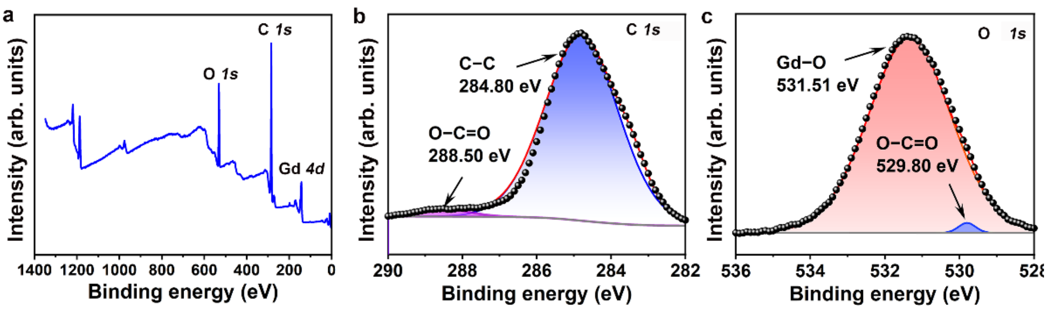

Supplementary Fig. S1 XPS spectra of SNWs. (a) XPS measurement spectra before ligand substitution of SNWs. High resolution XPS spectra of C *1s* (b) and O *1s* (c) peaks.

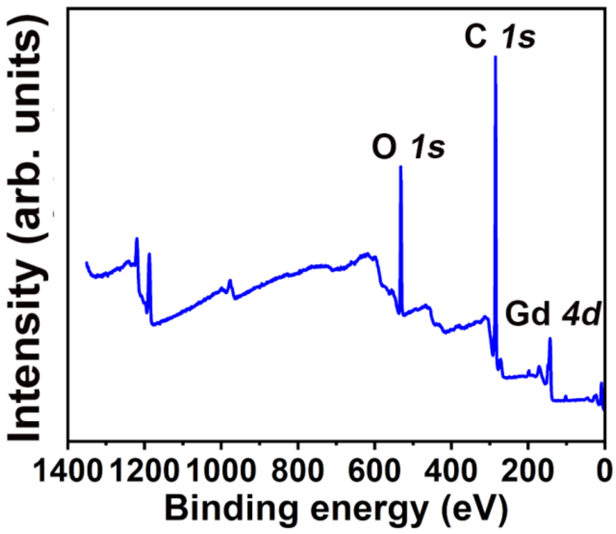

Supplementary Fig. S2 XPS measurement spectra after ligand substitution of SNWs.

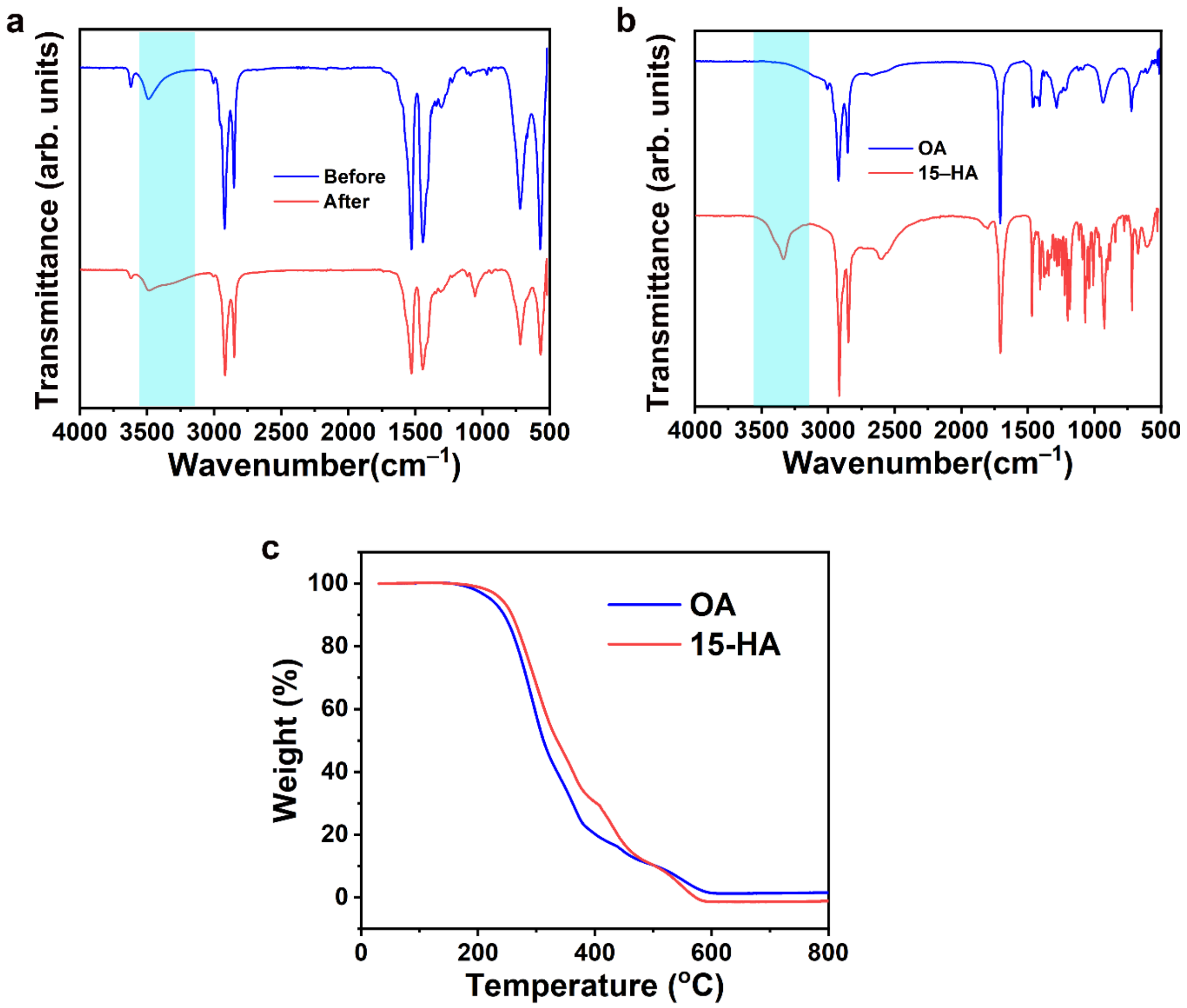

Supplementary Fig. S3 TG curve and FTIR curve. (a) FTIR curves of SNWs before and after ligand regulation. (b) FTIR curves of OA and after 15–HA. (c)Thermal decomposition curves of oleic acid (OA) and 15–hydroxypentadecanoic acid (15–HA).

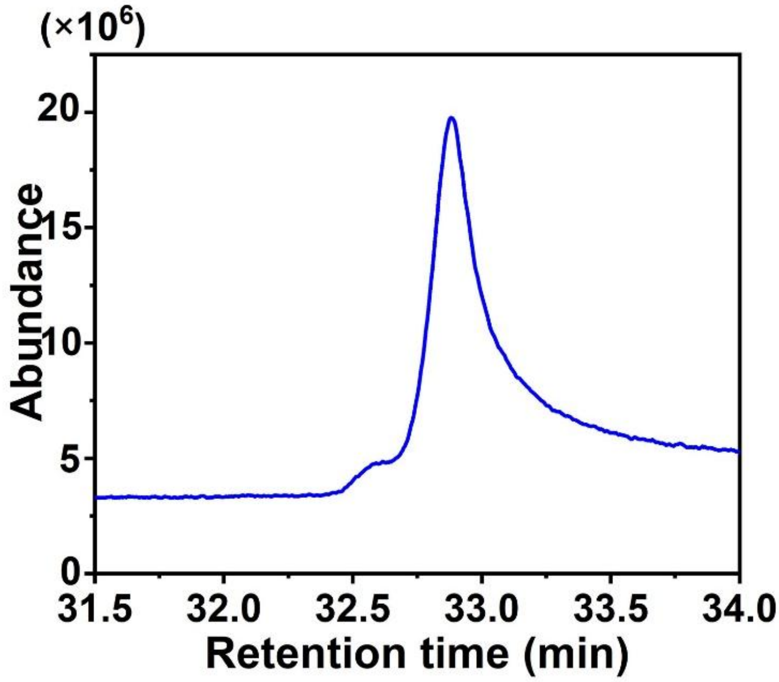

Supplementary Fig. S4 Gas chromatography of OA in solvent after ligand substitution.

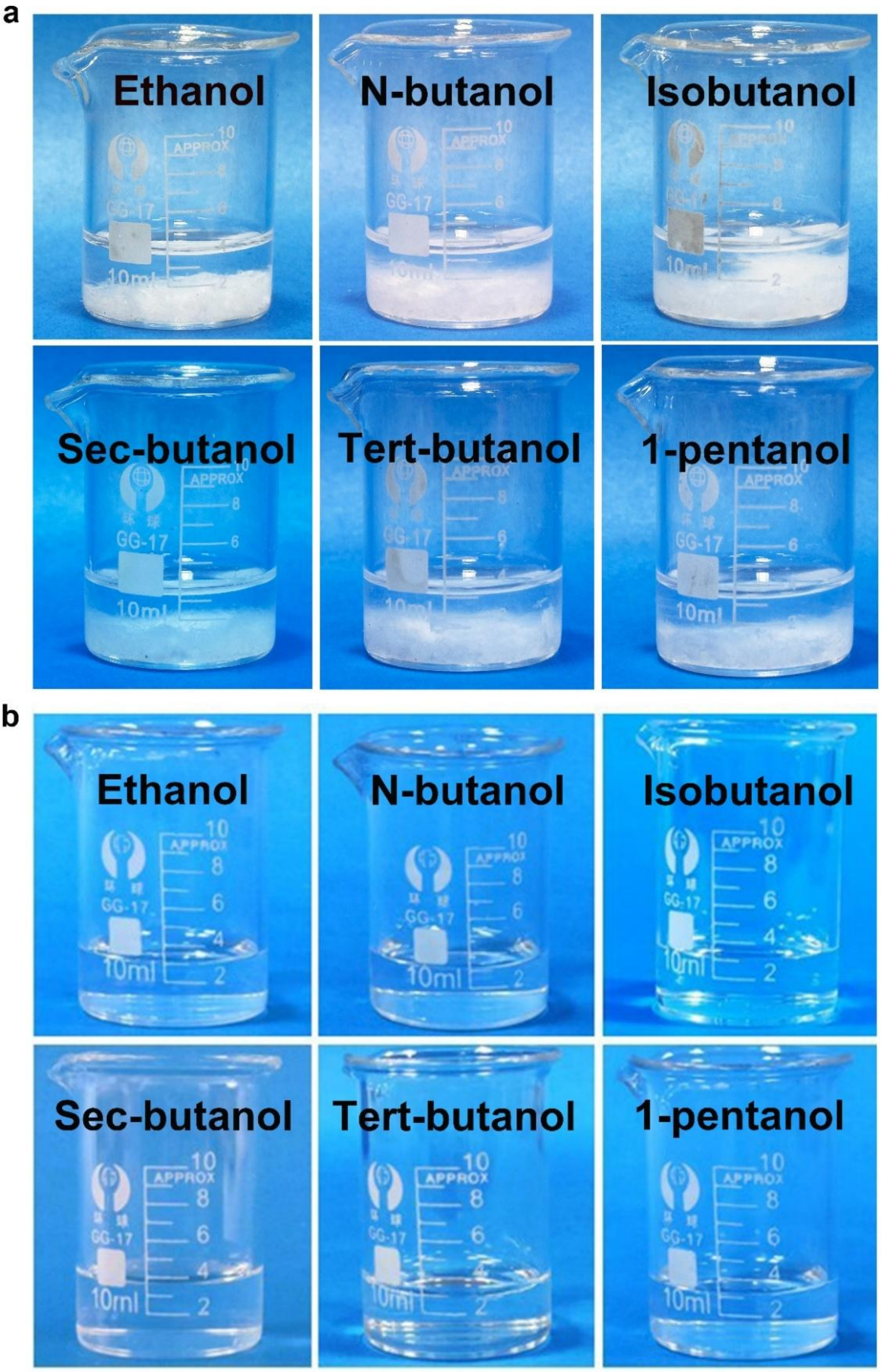

Supplementary Fig. S5 Dispersions of SNWs in ethanol, n-butanol, isobutanol, sec-butanol, tert-butanol, and 1-pentanol (a) before and (b) after ligand exchange.

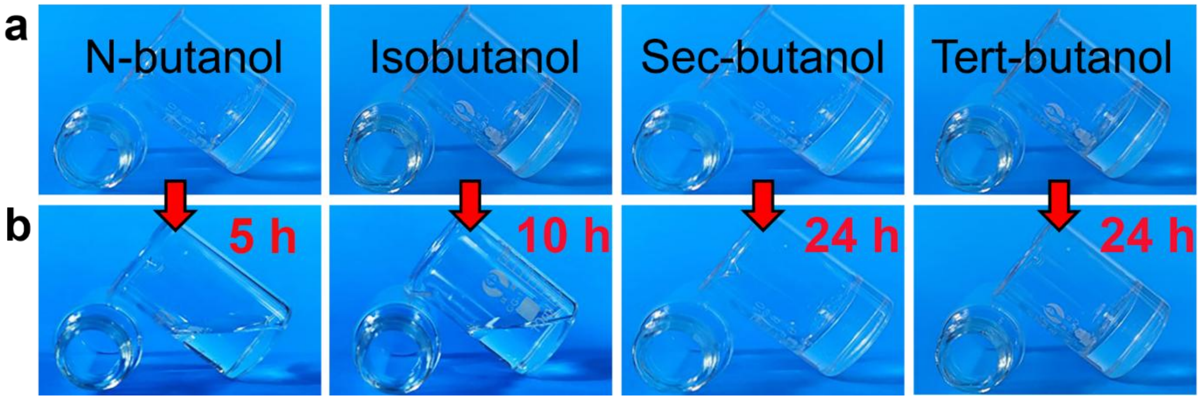

Supplementary Fig. S6 Gels of SNWs in butanol systems and their states at different times.

(a**)** Gel formed by SNWs in butanol system. (b) State of SNWs butanol gel after different time.

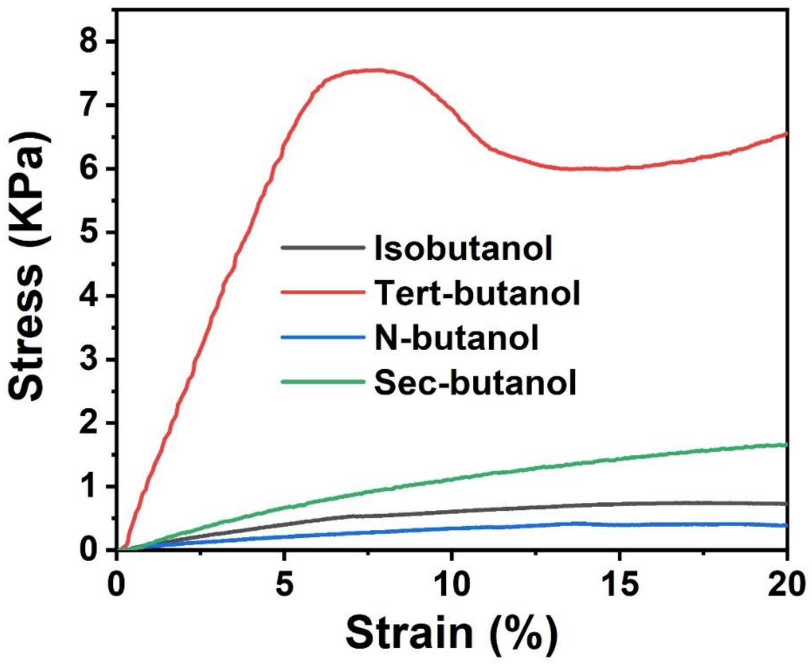

Supplementary Fig. S7 Stress strain curves of SNWs forming gel in butanol isomer solvent.

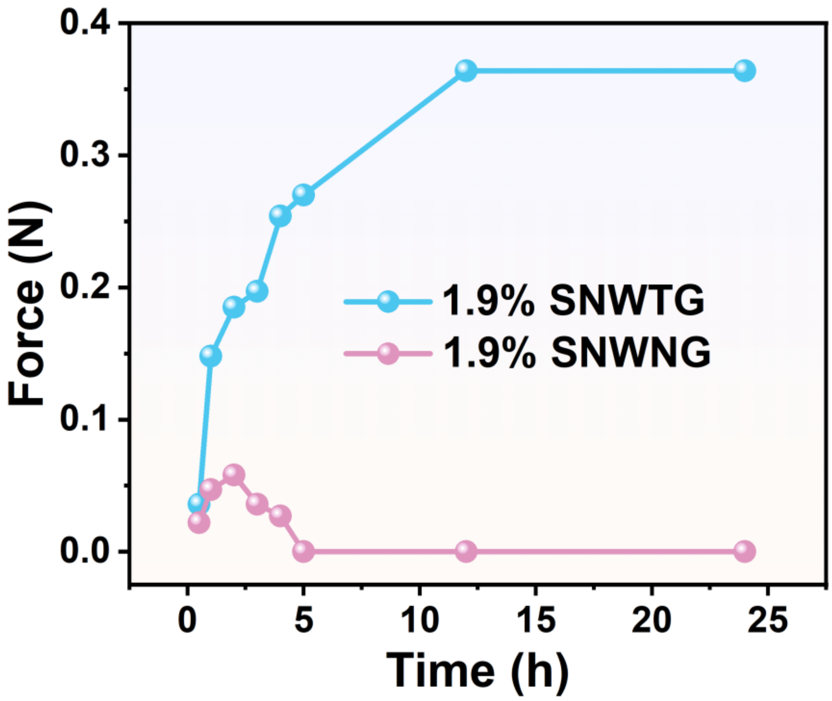

Supplementary Fig. S8 Localized pressure profiles of SNWNG and SNWTG over 24 h.

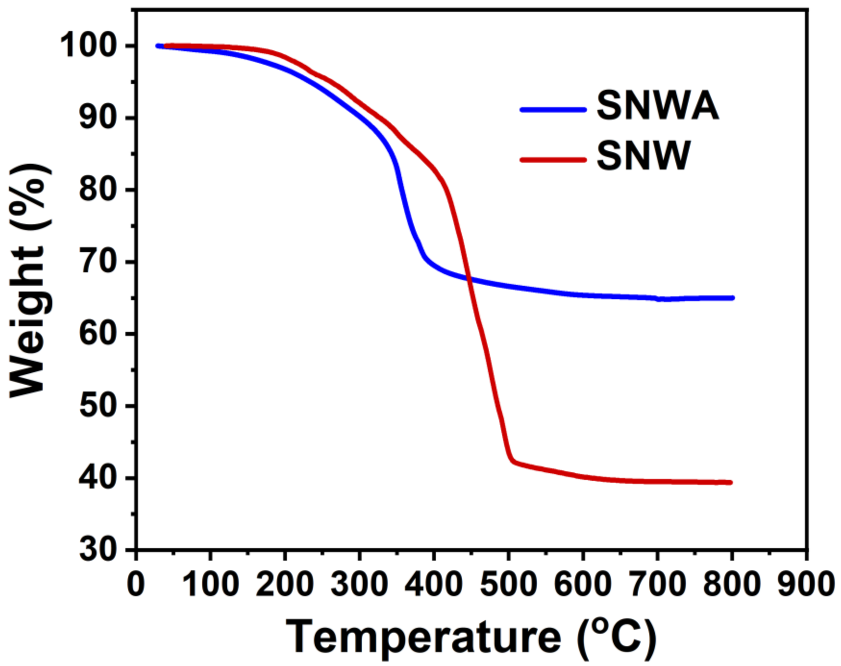

Supplementary Fig. S9 Thermal decomposition curves of SNW and SNWA.

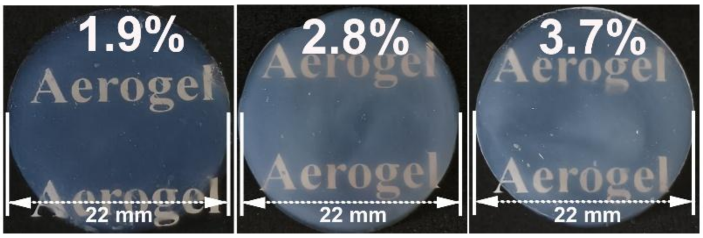

Supplementary Fig. S10 **SNWAs assembled in tert–butanol with different concentrations of SNWs.**

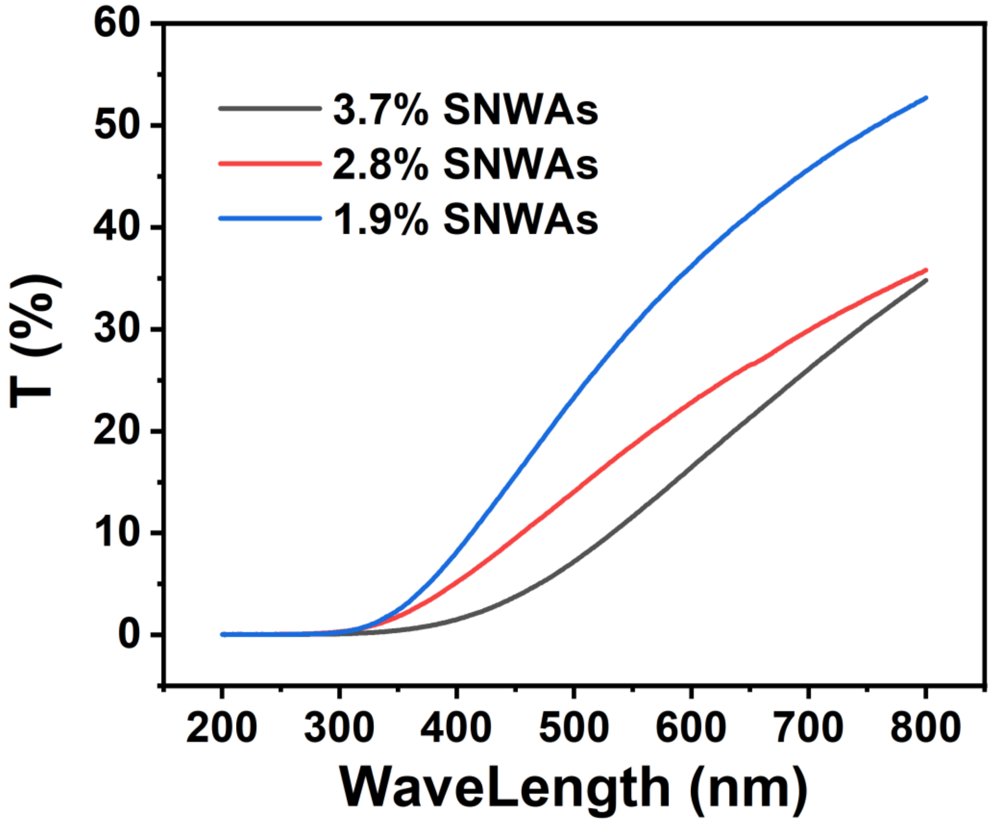

Supplementary Fig. S11 UV Vis Spectra of Aerogels Assembled with Gd–SNWs of Different Concentrations.

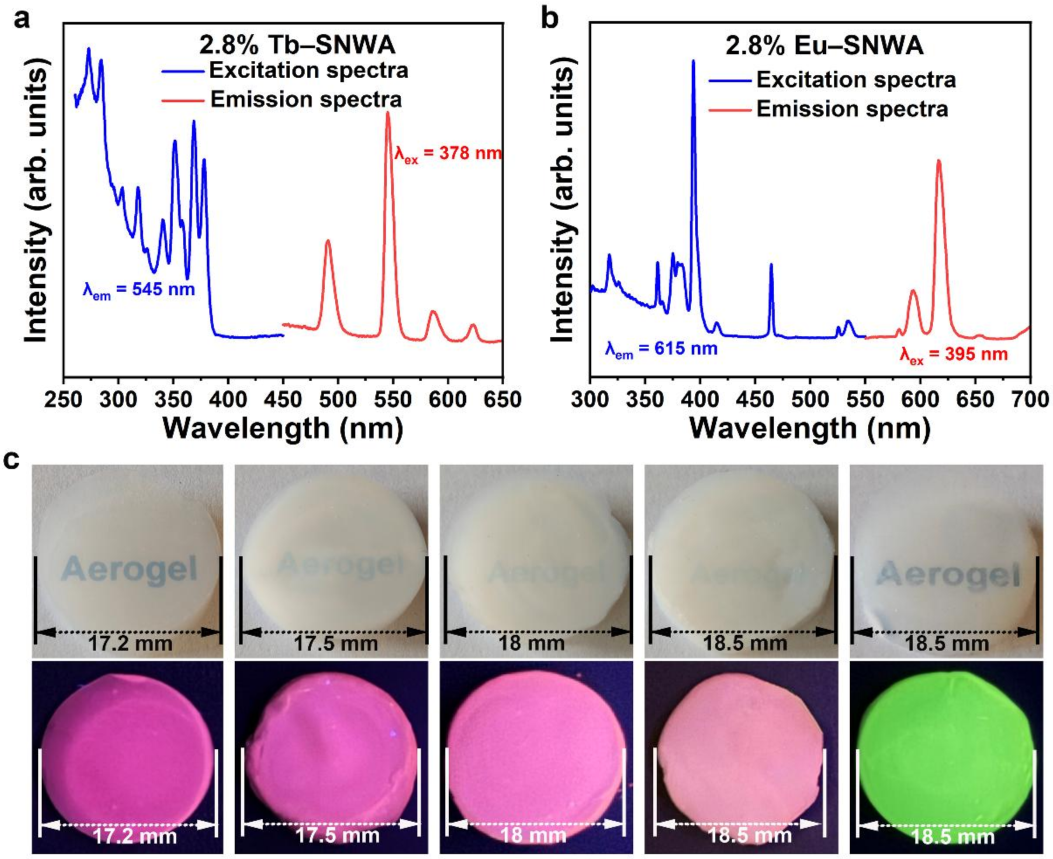

Supplementary Fig. S12. The PL spectrum of aerogel and its optical image under UV light. The PL excitation spectra and emission spectra of (a) Tb−SNWAs and (b) Eu−SNWAs. (c) SNWs gels and aerogels by mixing Eu−SNWs and Tb−SNWs with mass ratios of1:0, 7:33, 1:1, 3:7, and 0:1 (from left to right).

As illustrated in Supplementary Fig. S12b, the PL emission spectrum of the Eu−SNWA recorded under 395 nm excitation exhibits the characteristic transitions of Eu^{3+} ions from the $^5D_0$ excited state to the ^7F_J (J=1, 2, 3, 4) ground-state manifolds. The spectrum is dominated by the hypersensitive electric dipole transition at 615 nm ($^5D_0$-$^7F_2$), responsible for the intense red luminescence. The secondary peak at 590 nm corresponds to the magnetic dipole transition ($^5D_0$-$^7F_1$). Notably, the high intensity ratio of I($^5D_0$-$^7F_2$)/ I($^5D_0$-$^7F_1$) indicates that the Eu^{3+} ions

are situated in a low-symmetry coordination environment without inversion symmetry within the SNWA framework, which is beneficial for enhancing color purity.

The PL excitation spectra was recorded by monitoring the 615 nm emission. It consists of several sharp absorption bands in the 300–500 nm range, arising from the intra-configurational 4f-4f transitions of Eu^{3+}. The most prominent excitation peak is located at 395 nm ($^7F_0$-$^5L_6$), accompanied by other peaks such as 362 nm ($^7F_0$-$^5D_6$), 382 nm ($^7F_0$-^5G_J), and 465 nm ($^7F_0$-$^5D_2$). The strong absorption at 395 nm suggests that these Eu-SNWAs are highly compatible with near-UV excitation sources, making them promising candidates for advanced lighting and display technologies.

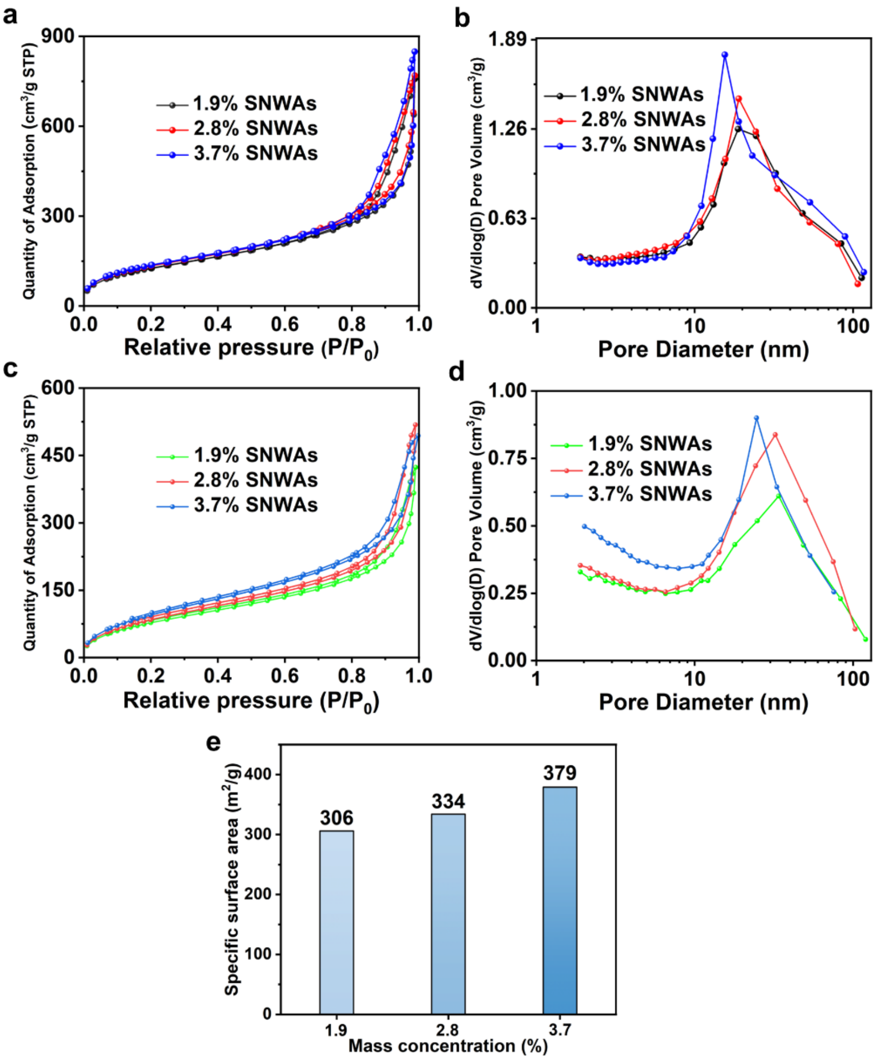

Supplementary Fig. S13 Analysis of specific surface area and pore size of aerogels. (a) Nitrogen adsorption-desorption isotherms and (b) Barrett-Joyner-Halenda (BJH) pore size distribution of Gd-SNWA. (c) Nitrogen adsorption-desorption isotherms, (d) BJH pore size distribution, and (e) specific surface area of silane-modified Gd-SNWA.

After silane modification, the nitrogen adsorption-desorption isotherm of Gd-SNWAs was identified as Type IV with a Type H1 hysteresis loop (Supplementary Fig. S13c), which confirms the preservation of a mesoporous structure within Gd-SNWAs. A comparison of the pore size distributions of SNWAs before and after silane modification revealed a remarkable

structural variation. First, a substantial reduction in the incremental pore volume was observed for all modified samples (Supplementary Fig. S13d), which directly demonstrates that silica species were deposited on the fibrous framework and partially blocked the internal void spaces. Second, the pore size range of modified SNWAs exhibited a slight shift toward larger values: the pore size peak of pristine SNWAs was centered at approximately 15–20 nm, whereas that of modified SNWAs shifted to the range of 25–40 nm. This phenomenon is attributed to the preferential blockage of smaller mesopores. These results are in excellent agreement with the observed reduction in the specific surface area of the aerogel after silane modification, which decreased from 505 m^2 g^{-1} to 379 m^2 g^{-1}.

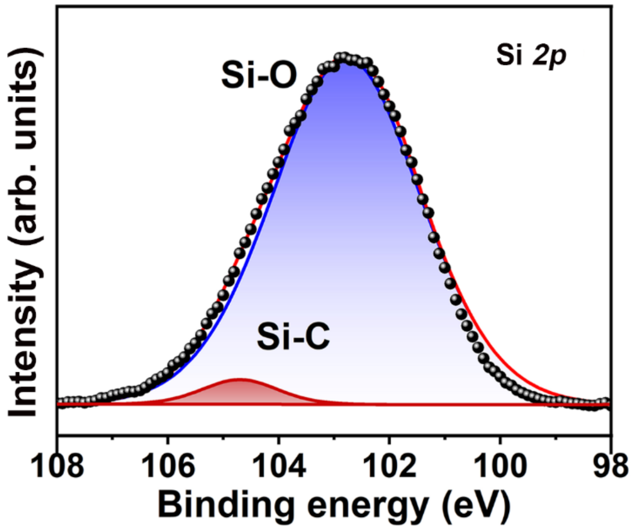

Supplementary Fig. S14 High-resolution XPS spectra of the Si 2p peak of aerogel after gas-phase deposition.

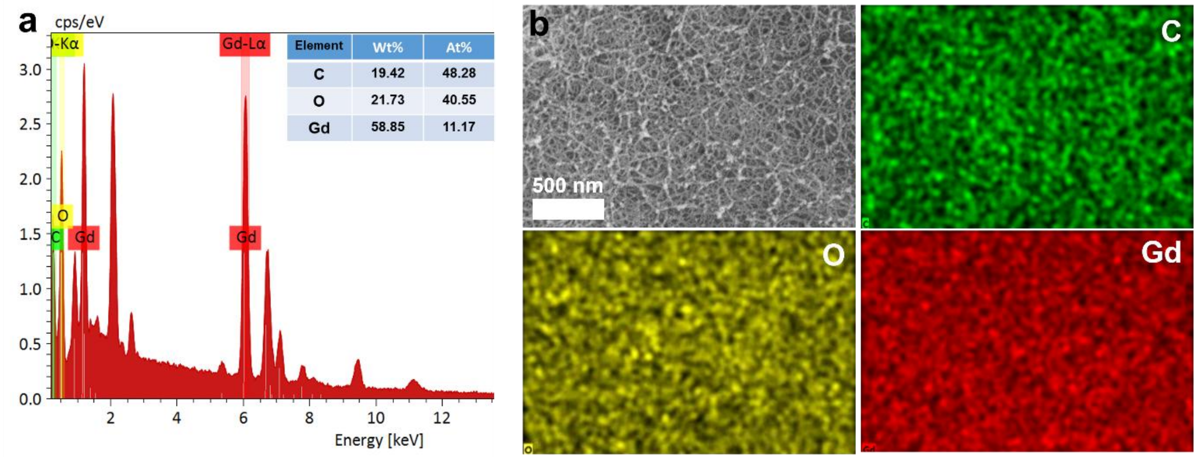

Supplementary Fig. S15 Energy dispersive spectroscopy of SNWAs. Element mapping (a), SEM image, and element distribution map (b) of SNWAs before silanization modification.

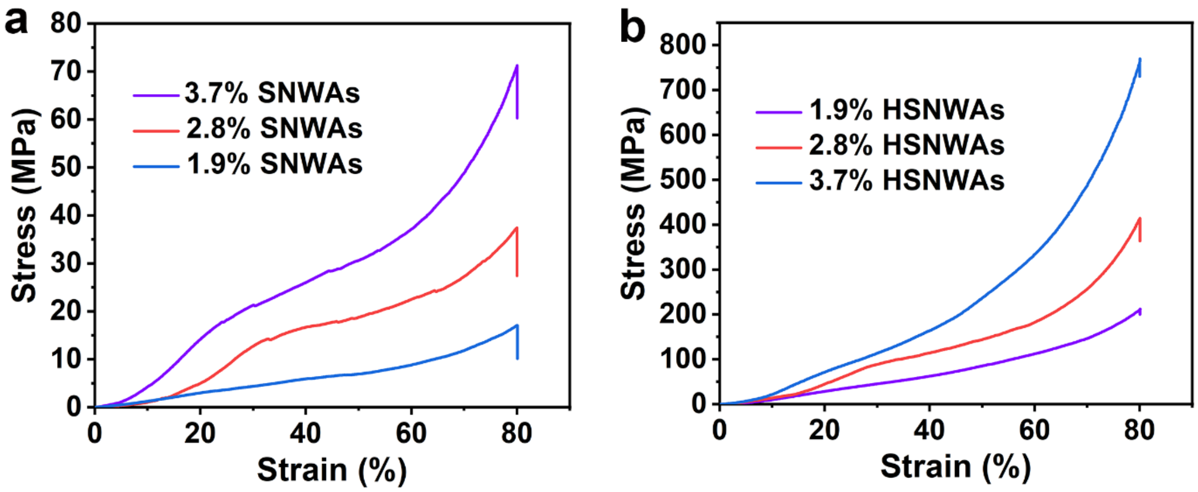

Supplementary Fig. S16 The stress-strain curves of SNWAs. The stress-strain curves of SNWAs assembled with different concentrations of SNWs (a) before and (b) after silane modification.

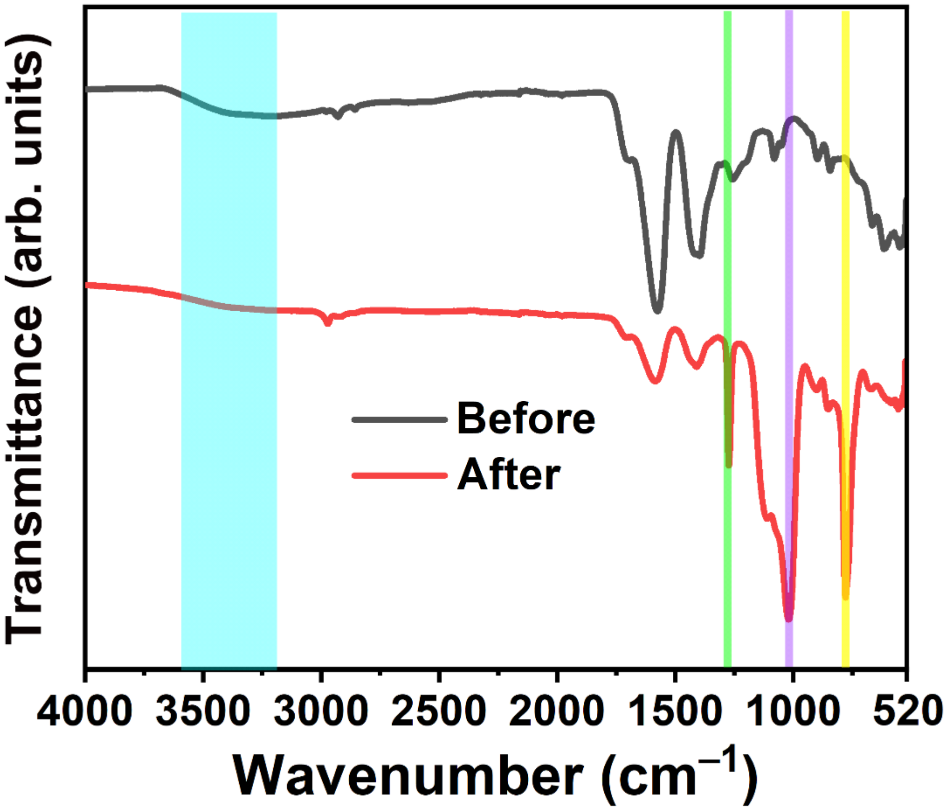

Supplementary Fig. S17 Fourier transform infrared spectra of SNWAs before and after silane modification.

References and Notes

1. Hess, B., Kutzner, C., van der Spoel, D. & Lindahl, E. GROMACS 4: Algorithms for Highly Efficient, Load-Balanced, and Scalable Molecular Simulation. *J. Chem. Theory Comp.* **4**, 435-447, (2008).
2. Essmann, U. et al. A smooth particle mesh Ewald method. *J. Chem. Phys.* **103**, 8577-8593, (1995).
3. Hess, B. et al., LINCS: A linear constraint solver for molecular simulations. *J. Comput. Chem.* **18**, 1463-1472, (1997).
