# Supplementary material for: Controllable assembly of sub-1 nm nanowires for the construction of aerogels
Source: Nat Commun. 2026 Mar 17;17:4053. doi: 10.1038/s41467-026-70713-8 (PMC13139467; doi:10.1038/s41467-026-70713-8)
Supplement: Supplementary file 2 — Description of Additional Supplementary Files [file 41467_2026_70713_MOESM2_ESM.pdf]

Description of Additional Supplementary Files

File Name: Supplementary Video 1
Description: MD simulation of assembly in SNWs dispersed systems after the addition of protons and ionized citrate molecules. The small blue and green models indicate an ionized citric acid molecule and proton, respectively.

File Name: Supplementary Video 2
Description: Compression testing of Gd-SNWAs under 50% strain.

File Name: Supplementary Video 3
Description: Fatigue resistance testing of silanized Gd-SNWAs under 50% strain.
